# The Microstructure Evolution Process and Flexural Behaviours of SiC Matrix Ceramic Infiltrated by Aluminium Base Alloy

**DOI:** 10.3390/ma15165746

**Published:** 2022-08-20

**Authors:** Wei Wang, Jinsheng Jia, Yong Sun, Zhuang Kong, Tianyi Na, Liangliang Yang, Ruina Ma, Qiang Li

**Affiliations:** 1 State Key Laboratory of Green Building Materials, China Building Materials Academy, Beijing 100024, China; 2 School of Materials Science and Engineering, Hebei University of Technology, Tianjin 300130, China

**Keywords:** Ti_3_Si(Al)C_2_, SiC, infiltration, microstructure, flexural behaviours

## Abstract

In this paper, an infiltration approach was proposed to generate a Ti_3_Si(Al)C_2_ transition layer in SiC matrix composites to effectually strengthen SiC ceramics. The infiltration temperature played a significant role in the evolution of the microstructure, phase composition, and flexural behaviours. Molten aluminium base alloy fully penetrated SiC ceramic after infiltration at different experimental temperatures (800–1000 °C). The phases in the reaction layer on the surface of SiC ceramic samples varied with the infiltration temperature. When infiltrated at 800 °C, only SiC and Al phases can be found in SiC composites, whereas at 900 °C, a reaction layer containing Ti_3_Si(Al)C_2_ and SiC was produced. The Ti_3_Si(Al)C_2_ phase grew in situ on SiC. At 1000 °C, the Ti_3_Si(Al)C_2_ phase was unstable and decomposed into TiC and Ti_5_Si_3_. The cermet phase Ti_3_Si(Al)C_2_ was synthesized at a relatively low temperature. Consequently, the flexural modulus and three-point bending strength of samples infiltrated at 900 °C was enhanced by 1.4 and 2.4 times for the original SiC ceramic, respectively.

## 1. Introduction

In recent times, Ti_3_SiC_2_ has been regarded as the most promising novel ceramic material. Ti_3_SiC_2_ is a type of MAX phase (a group of ternary compounds where M is a transition metal, A is an A-group element, and X is either C or N). It has a hexagonal crystal structure (space group *P*6_3_/*mmc*) and has a theoretical density of 4.53 g/cm^3^ [1,2]. It possesses an unusual combination of metal and ceramic properties, such as high melting temperature, high electrical and thermal conductivity, low density, and excellent resistance to oxidation and thermal shock. It is also readily machinable and has exceptional damage tolerance [3,4,5,6,7,8,9,10,11]. 

Since Jeitschko et al. first synthesized Ti_3_SiC_2_ in 1967 through a chemical reaction between TiH_2_, Si, and graphite, numerous methods for the synthesis of Ti_3_SiC_2_ have been reported. Foratirad et al. [12] synthesized nanolayered Ti_3_SiC_2_ MAX phase via infiltration at 1500 °C of porous TiC by the gel casting process. The maximum content of Ti_3_SiC_2_ of 85 wt% and the lowest hardness of 6 GPa were obtained. Amiri et al. [13] fabricated Ti_3_SiC_2_-SiC composites by hot-pressing Ti_3_SiC_2_ and SiC powders under a pressure of 35 Mpa in a vacuum environment at 1550 °C and obtained an enhanced Vikers hardness of 13.9 Gpa. Afterwards, they prepared the Ti_3_SiC_2_-SiC composites by reactive hot-pressing TiC and the Si precursor [14]. The composites exhibited the highest Vickers hardness of 14.2 GPa, a fracture toughness of 10.1 MPa/m^2^, and a flexural strength of 576 MPa. Csáki et al. [15] prepared the Ti_3_SiC_2_ phase from Ti, TiC, and SiC using spark plasma sintering. A maximum MAX phase content of ~90 wt% and the highest Vickers hardness were achieved at the sintering temperature of 1550 °C and 1450 °C, respectively. Yang et al. [16] synthesized Ti_3_SiC_2_ film by molten salt synthesis route of Ti and Si powder based on polymer-derived SiC fibre substrate. The Ti_3_SiC_2_ film was utilized as an interphase to prepare the SiC fibre reinforced SiC matrix composites and obtained the flexural strength of 460 ± 20 MPa and fracture toughness of 16.8 ± 2.4 MPa/m^2^. In addition, reactive melt infiltration, pressureless sintering, or hot isostatic pressing were also used to prepare Ti_3_SiC_2_ [17,18,19]. However, these methods are limited by efficiency, cost, controllability, complexity, and severe processing conditions, e.g., high temperature (e.g., 1400 °C), vacuum, and protective gas [13,20].

Various studies have demonstrated that Ti_3_Si(Al)C_2_ (where some of the Si atoms in Ti_3_SiC_2_ are substituted by Al atoms) possesses similar mechanical properties to Ti_3_SiC_2_, and even exhibits better oxidation resistance [21,22,23,24]. Compared with Ti_3_SiC_2_, Ti_3_Si(Al)C_2_ can be generated by reactive melt infiltration at lower temperatures [21]. In our previous work [3], we reported a novel method for generating Ti_3_Si(Al)C_2_ by melt infiltration from a SiC ceramic base material. This method displayed many advantages, such as a simple processing route and a low processing temperature, and notably improved the mechanical properties. However, the effect of infiltration temperature on the microstructure evolution in the reaction layer and the reaction mechanism of Ti_3_Si(Al)C_2_ were not completely confirmed. Hence, in the current study, we prepared a low-melting-point aluminium base alloy which was able to wet the SiC ceramic, and studied the effect of infiltration temperature on the microstructure evolution in the reaction layer and the formation mechanism of Ti_3_Si(Al)C_2_. 

## 2. Experimental Procedure

The original SiC ceramic (3.02 g/cm^3^, Huamei Ceramics Co., Ltd., Weifang, China) is composed of SiC and Si phases. It has an open porosity of 1.48%, with small pores distributed in the gaps of SiC and Si. The SiC ceramic was cut into 5 mm × 5 mm × 10 mm and 5 mm × 5 mm × 40 mm samples using electro-discharge machining, and then polished using a diamond grinding disc up to grade 1500. The specimens were cleaned ultrasonically in acetone and alcohol for 10 min, respectively. The Al base alloy was prepared in a vacuum furnace from Al (99.5%, Fuxin Jinshu Co., Ltd., Baoji, China) and Ti ingots (99.9%, Fuxin Jinshu Co., Ltd., Baoji, China), silicon (99.5%, PRAM Co., Ltd., Beijing, China), and Cu particles (99.999%, PRAM Co., Ltd., Beijing, China). The stoichiometric composition was 76.5 wt% Al, 8.5 wt% Ti, 10 wt% Si, and 5 wt% Cu. The melt infiltration procedure was carried out by first melting the Al base alloy in an electric furnace, and then placing the SiC specimens into the molten alloy. The infiltration temperature and time were changed to determine the optimal processing conditions. 

The morphologies of the specimens were observed by scanning electron microscopy (SEM, Nova Nano SEM450, FEI Co., Hillsboro, OR, USA) and transmission electron microscopy (TEM, Tecnai G2 F20 S-TWIN, FEI Co., Hillsboro, OR, USA). The preparation process of transmission pattern is as follows. First, a 5 mm × 5 mm × 1 mm sample was cut by wire cutting, and then polished by a diamond grinding disc to the thickness of 40–50 μm. Finally, the sample was polished by Gatan precision ion polishing system. The chemical composition was examined by using an energy dispersive X-ray spectroscopy (EDS). The phases of the specimens were identified by X-ray diffraction (XRD, Dmax 2500, Rigaku, Tokyo, Japan) using a Cu Kα radiation source, for 2θ = 10–90°. The element composition and chemical state of the samples were analyzed by X-ray photoelectron spectroscopy (XPS, ESCALAB 250Xi, Thermo Scientific, Waltham, MA, USA).

Differential scanning calorimetry (DSC) experiments were carried out in a thermal analyser (SDT Q-600, TA Instruments, New Castle, DE, USA) to determine the heat change of the samples. Mixture of fine solid particles of Al base alloy and SiC ceramic with a total weight of about 10 mg and fine solid particles of Al base alloy with a total weight of about 10 mg were placed into Al_2_O_3_ crucibles, respectively, and then heated from an ambient temperature to 1300 °C at a rate of 10 °C/min under Ar flow.

Infiltrated SiC ceramic specimens of 5 mm × 5 mm × 40 mm were prepared for three-point-bending tests (CMT6104, Xinsansi Co., Shenzhen, China) to determine the bending strength and flexural modulus at room temperature, wherein specimens with a 30 mm span were subjected to a crosshead speed of 0.6 mm/min. Five samples were measured for each set of experimental conditions, in order to ensure the reliability of the experimental results.

## 3. Results and Discussion

### 3.1. Effect of Infiltration Time on the Microstructure of SiC Matrix Composites

To investigate the microstructure evolution of the SiC matrix composites, the effect of infiltration holding time were studied. During the infiltration procedure, SiC ceramic samples were immersed in a molten Al base alloy and then the Al base alloy infiltrated the SiC ceramic. 

**Figure 1:**
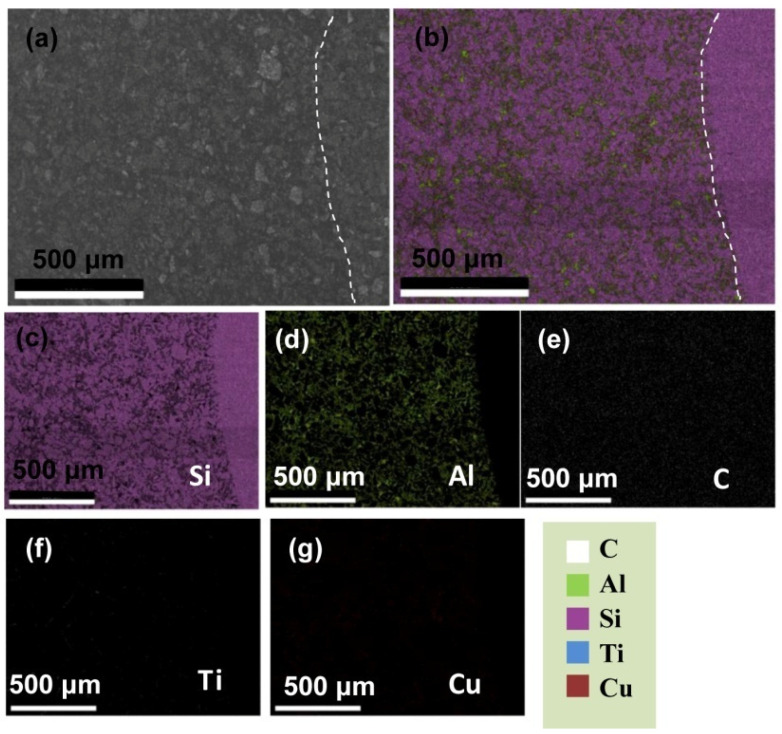
SEM image and element maps of interface of infiltration (**b**–**g**) and no infiltration (**a**).

Figure 1 shows the SEM and corresponding element distribution map of the infiltration and no infiltration parts of SiC matrix ceramics. From Figure 1a,b, it can be seen that the left side of the dashed line is the SiC ceramic sample with melted Al base alloy, and the right side is the part without alloy. There are very obvious differences between the two parts. As shown in Figure 1c, purple represents the distribution of element Si. The Si distributed relatively uniform at the right side without the Al base alloy, because the SiC matrix ceramic is mainly composed of SiC particles and Si embedding in the gaps of SiC particles. However, the purple Si elements mainly present as irregular and blocky in the infiltrated part, and the gaps of SiC particles present as dark. Combined with Figure 1d, the green color represents the Al element distribution, and it can be found that the green color mainly distributes in the left part infiltrated the Al base alloy and does not exist in the right part without the Al base alloy. Moreover, from the green color distribution, it can be seen that Al elements are mainly distributed around the irregular mass around. Therefore, it can be inferred that Al elements are mainly distributed in the gaps of SiC particles during the infiltration process. Figure 1e–g are the distribution maps of C, Ti, and Cu elements, respectively. However, the color of Ti and Cu are rarely collected, indicating that the contents of Ti and Cu in the SiC matrix composites are relatively low through the infiltration process. 

**Figure 2:**
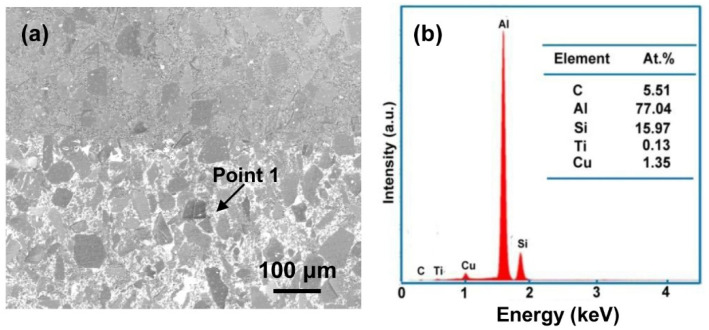
(**a**) SEM image of no infiltration (up) and infiltration (down), (**b**) EDS analysis of Point 1.

Figure 2a shows the image of no infiltration (up) and infiltration (down). It is obvious that Al alloy infiltrated the SiC matrix ceramics exists at the gaps of SiC particles. According to the EDS analysis of Point 1 from Figure 2b, the ratio of Si and Al is higher than the Al base alloy. It can be inferred that the Al base alloy dissolved the Si existing in the gaps of the SiC particles and then penetrated the SiC ceramic sample. It also can be found that the content of Ti and Cu is low, and corresponds to the element maps in Figure 1.

**Figure 3:**
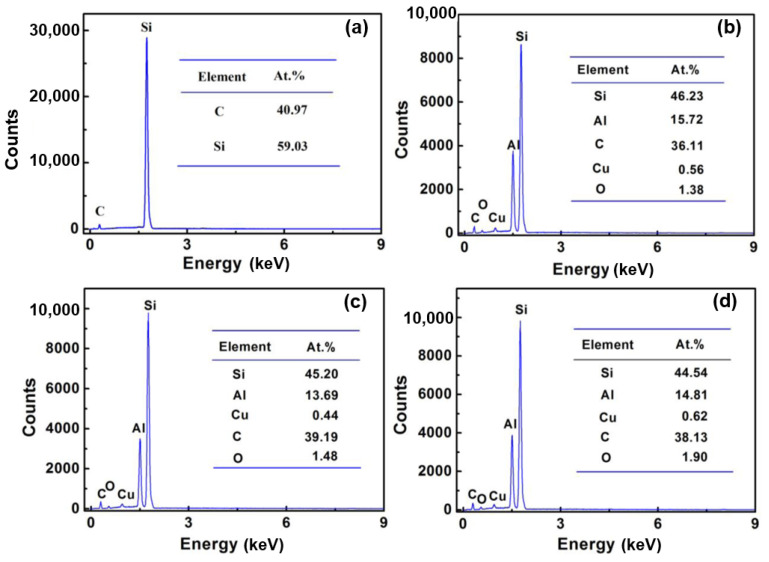
EDS analysis of SiC ceramic inner areas infiltrated at 800 °C for different time: (**a**) SiC ceramic, (**b**) 30 min, (**c**) 2 h, and (**d**) 6 h.

Through analysing the Figure 1 and Figure 2, it is found that the distribution of Al elements can be used to determine whether the alloy infiltrates SiC ceramics. The content of Al can be detected when it infiltrates the SiC ceramic sample. Accordingly, Figure 3 shows the EDS analysis of the sample centre infiltrated at 800 °C for a different time. Figure 3a shows that original SiC ceramic mainly contains 40 at.% C and 60 at.% Si. Figure 3b–d exhibit the EDS analysis of the sample centre infiltrated at 800 °C for 30 min, 2 h, and 6 h, respectively. The corresponding Al content was 15.72 at.%, 13.69 at.% and 14.81 at.%. It can be inferred that melting Al can infiltrate the SiC ceramic sample with 5 mm thickness completely when the infiltration time is above 30 min at 800 °C.

**Figure 4:**
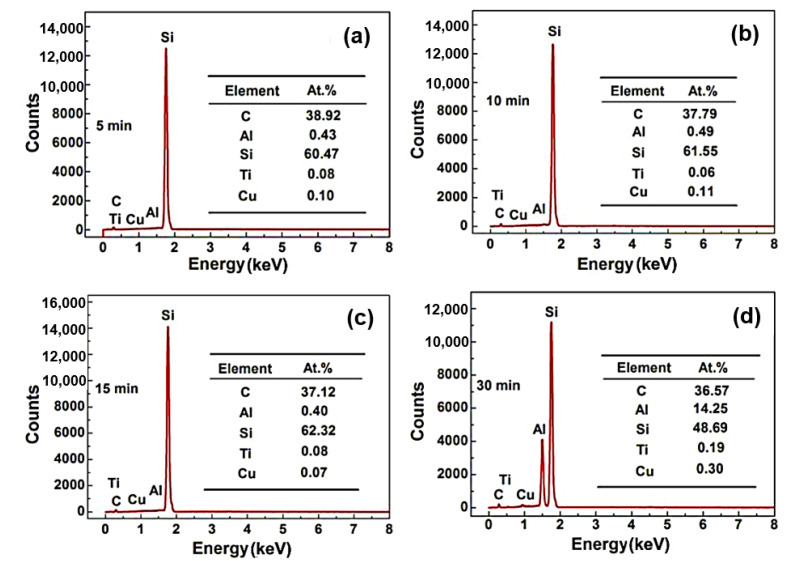
EDS analysis of SiC ceramic inner areas infiltrated at 900 °C for different time: (**a**) 5 min, (**b**) 10 min, (**c**) 15 min, and (**d**) 30 min.

Figure 4 shows the EDS analysis of SiC ceramic sample centre infiltrated by the Al base alloy at 900 °C for different times (5 min, 10 min, 15 min, 30 min). As seen from Figure 4a–c, the centre of sample mainly contains C, Si elements, and trace amounts of Al, Ti, and Cu. Figure 4d shows the sample centre after being infiltrated for 30 min and containing 14.25 at.% Al, 36.57 at.% C, and 48.69 at.% Si. It is noted that the centre of the sample does not contain the Al element when the infiltration time is less than 15 min, which indicates that the alloy does not infiltrate the sample centre. From the above analysis, the sample with 5 mm thickness would be completely infiltrated by the alloy after 30 min at 800 °C and 900 °C. Therefore, it can be inferred that the sample with 5 mm thickness could be infiltrated completely under an infiltration condition with a temperature higher than 800 °C and a time longer than 30 min.

The porosity of the original SiC ceramic is 1.48 vol.%, measured by the Archimedes method [3]. Molten Al base alloy could infiltrate into SiC ceramic samples with 5 mm thickness at 800 °C, 900 °C and 1000 °C for a time longer than 30 min. It is obviously different from traditional impregnation relying on the open porous. Melton Al alloy made contactwith the Si, embedding in the gaps of SiC particles, and then dissolving Si gradually. The channels for Al alloy infiltrating into SiC samples were built, therefore the Al alloy can infiltrate the SiC sample.

### 3.2. Effect of Infiltration Temperature on the Microstructure of SiC Matrix Composites

**Figure 5:**
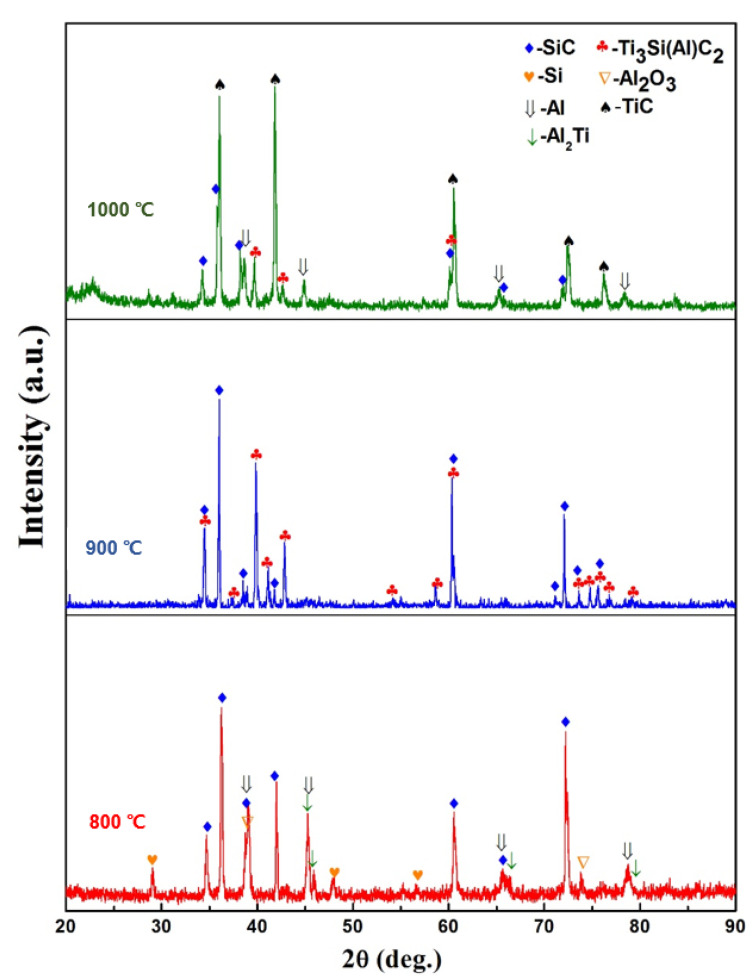
XRD patterns of SiC ceramics infiltrated at 800, 900, and 1000 °C for 6 h.

Figure 5 shows XRD patterns of the surface of SiC ceramic that infiltrated the Al base alloy at 800, 900, and 1000 °C for 6 h. After infiltration at 800 °C, there was no obvious change to the SiC structure. The surface of the SiC ceramic contains mainly SiC and Al, with a small amount of oxides and Si. As the infiltration temperature increased to 900 °C, diffraction peaks of Ti_3_Si(Al)C_2_ at 2*θ* = 34.13°, 39.58°, 42.55°, and 60.33° became more intense, with that of SiC simultaneously decreasing. After infiltration at 1000 °C, diffraction peaks of TiC at 2*θ* = 35.89°, 41.68° were more intense than those of the SiC and Ti_3_Si(Al)C_2_ phases, indicating that more TiC was generated.

**Figure 6:**
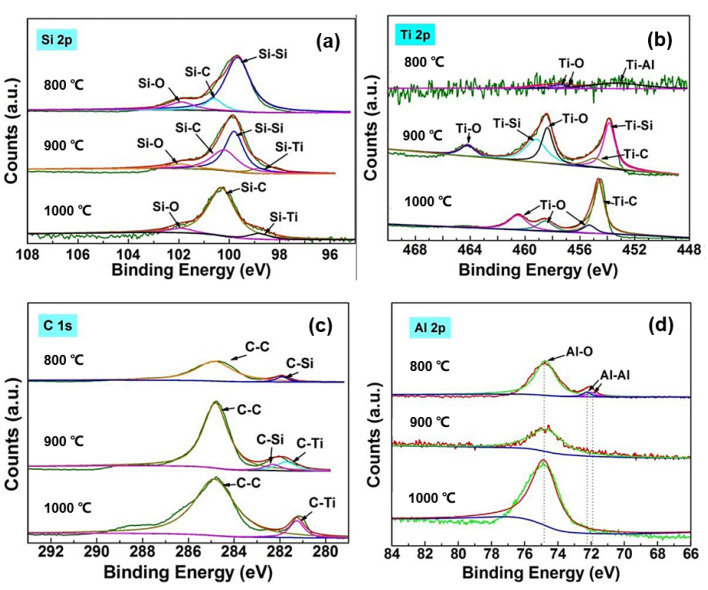
XPS spectra of the Si 2p (**a**), Ti 2p (**b**), C 1s (**c**), and Al 2p (**d**) lines of SiC ceramics infiltrated at 800, 900, and 1000 °C for 6 h.

The XRD analysis implies that the constituent phases in the reaction layer change significantly when the infiltration temperature changes. To explore this in more detail, elemental analyses were carried out using X-ray photoelectron spectroscopy (XPS). As shown in Figure 6a, the Si 2p spectrogram of samples infiltrated at 800 °C for 6 h mainly consists of three peaks (99.7, 100.6, and 101.9 eV), corresponding to the Si–Si bond of Si, Si–C bond of SiC, and Si–O bond of SiO_x_, respectively. The samples infiltrated at 900 °C for 6 h gave mainly four peaks (98.8, 99.8, 100.2, and 101.9 eV), corresponding to the Si–Ti bond of TiSi_2_, Si–Si bond of Si, Si–C bond of SiC, and Si–O bond of SiO_x_, respectively. The samples infiltrated at 1000 °C had similar peaks to that at 900 °C, but no peak corresponding to the Si–Si bond was present. The Ti 2p spectra in Figure 6b for the samples infiltrated at 800 °C consist of three peaks (453.3, 457.5, and 458.9 eV). The former peak corresponds to the binding energy of Ti–Al of Al_3_Ti, and the latter two peaks correspond to the energy of the Ti–O bond in TiO_2_. The samples infiltrated at 900 °C displayed five peaks (453.86, 454.89, 458.37, 459.1, and 464.19 eV), which correspond to the Si–Ti, Ti–C, Ti–O, Si–Ti, and Ti–O bonds. Infiltration at 1000 °C gave four peaks (454.6, 455.3, 458.4, and 460.51 eV). The former peak corresponds to the Ti–C bond in TiC, and the latter three peaks correspond to the Ti–O bond in TiO_2_. The C 1 s spectra in Figure 6c demonstrates that all the samples included the C–C bond. After infiltration at 800 °C, the main peak is of the C–Si bond, whereas those after infiltration at 900 °C were of C–Si and C–Ti bonds. At 1000 °C, the C–Ti bond dominates. The Al 2p spectra in Figure 6d shows that the main bonds in the samples infiltrated at 800 °C are Al–O and Al–Al, whereas after infiltration at 900 or 1000 °C, the major bond is of Al–O alone. The XPS results demonstrate that there are slight oxides on the surface. The major phases of the composites are SiC and Al after 800 °C infiltration, SiC and Ti_3_SiC_2_ after 900 °C infiltration, and SiC and TiC after 1000 °C infiltration, which coincide with the results of the XRD phase analysis in Figure 5.

The effects of infiltration temperature can be clearly seen from the above analysis. After infiltration at 800 °C, the diffraction peaks revealed that there was no formation of Ti_3_Si(Al)C_2_, whereas at 900 °C, Ti_3_Si(Al)C_2_ formed obviously. As the infiltration temperature increased further to 1000 °C, diffraction peaks of TiC appeared, and the intensity of Ti_3_Si(Al)C_2_ decreased.

**Figure 7:**
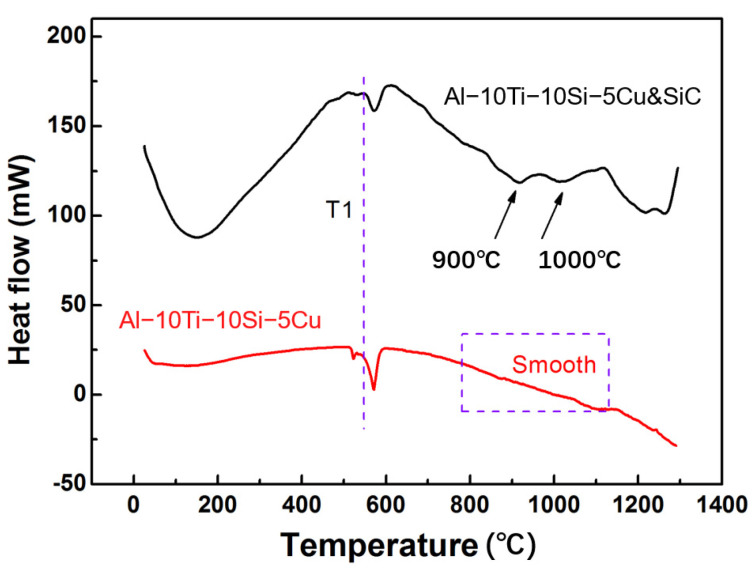
DSC analyses of mixtures of Al base alloy and SiC ceramic.

Previously, El-Raghy [11] and Harimkar [25] reported that the intermediate phases in the formation of Ti_3_SiC_2_ (from Ti/SiC/C powders) were TiC_x_ and Ti_5_Si_3_C_x_. Using 3Ti/1Si/2C powder, Zhang et al. found that Ti_5_Si_3_C_x_ formed at about 820 °C, and TiC_x_ formed at about 980 °C, by DSC, XRD, and EDS analyses [26]. Hence, we performed DSC analyses on mixtures of Al base alloy and SiC ceramic (Figure 7) to determine the formation temperatures of phases in the infiltrated samples. An endothermic peak at 570 °C and exothermic peaks starting at about 900 and 1000 °C can be observed. In the DSC curve of the Al base alloy, only the endothermic peak appears at about 570 °C and hence it can be inferred to be the melting point of the Al base alloy, noting that the composition of Al is 76.5 wt.%. Feng et al. [27] reported that the reaction to form Ti_3_SiC_2_ is exothermic. Hence, by combining the results with those of XRD and EDS, the exothermic peak starting at 900 °C was assigned to the formation of Ti_3_Si(Al)C_2_. The lack of peaks around 820 °C show that the formation process of Ti_3_Si(Al)C_2_ is different from that in the works of El-Raghy [11] and Harimkar [25], and therefore that TiC_x_ and Ti_5_Si_3_C_x_ were not intermediate phases in the formation of Ti_3_SiC_2_ from infiltrating molten Al base alloy into SiC ceramic.

**Figure 8:**
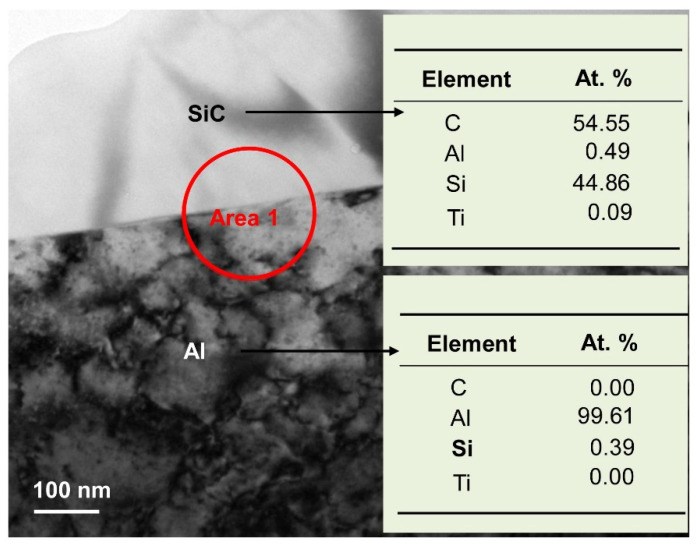
Cross-sectional TEM image with EDS analysis of the SiC–Al interface.

**Figure 9:**
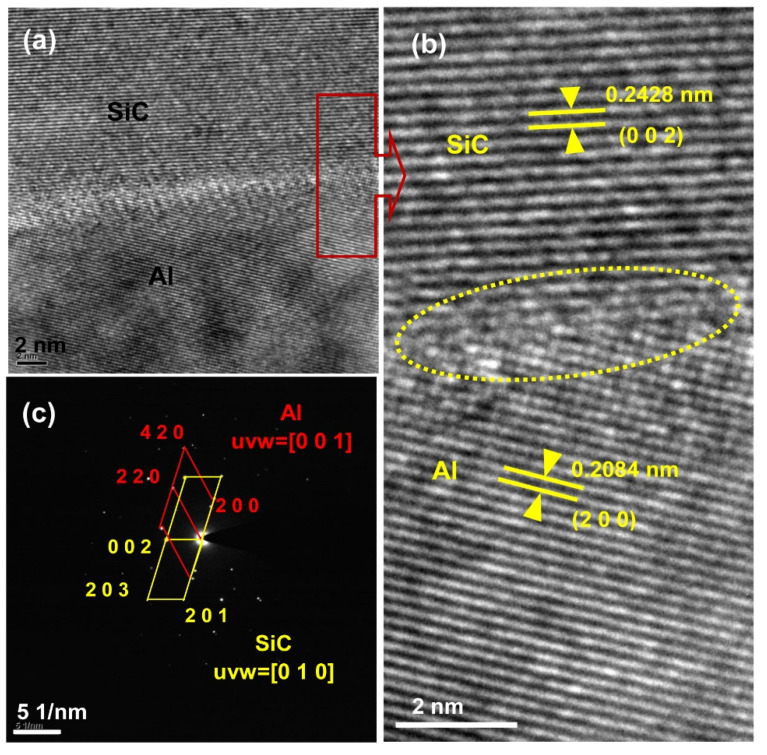
HRTEM images (**a**,**b**) and SAED patterns (**c**) of the SiC–Al interface.

To obtain more information on the microstructures, cross sectional TEM and EDS analyses were performed, as shown in Figure 8. SiC was seen to coexist with Al, which is consistent with the XRD results (Figure 5). HRTEM images and SAED patterns of area 1 (from Figure 8) are shown in Figure 9. SiC phase and Al phase are evidenced by the HRTEM images in Figure 9a,b; high magnification micrographs of the interface between SiC (ICDD PDF: 48-0708, hexagonal, *a* = 0.3081 nm, *c* = 0.503 nm) and Al (ICDD PDF: 04-0787, cubic, *a* = 0.4049 nm) are shown in Figure 9b. Infiltration at 800 °C produced an incoherent interface, which implies that the interfacial energy is large, and the formation of new phases at the interface is difficult. About three atomic layers at the interface occurred dislocation and some vacancies occurred in the interface of Al and SiC phases, but the Al phase is in contact with the SiC phase. The corresponding SAED pattern (shown in Figure 9c) reveals no other compounds formed at the interface, which is in good agreement with the XRD results. Therefore, Al alloy dissolved Si existing in the SiC particles gap gradually, and the channels for Al alloy infiltrating SiC samples were built. Then, the Al alloy can infiltrate the SiC sample without new phase formation.

**Figure 10:**
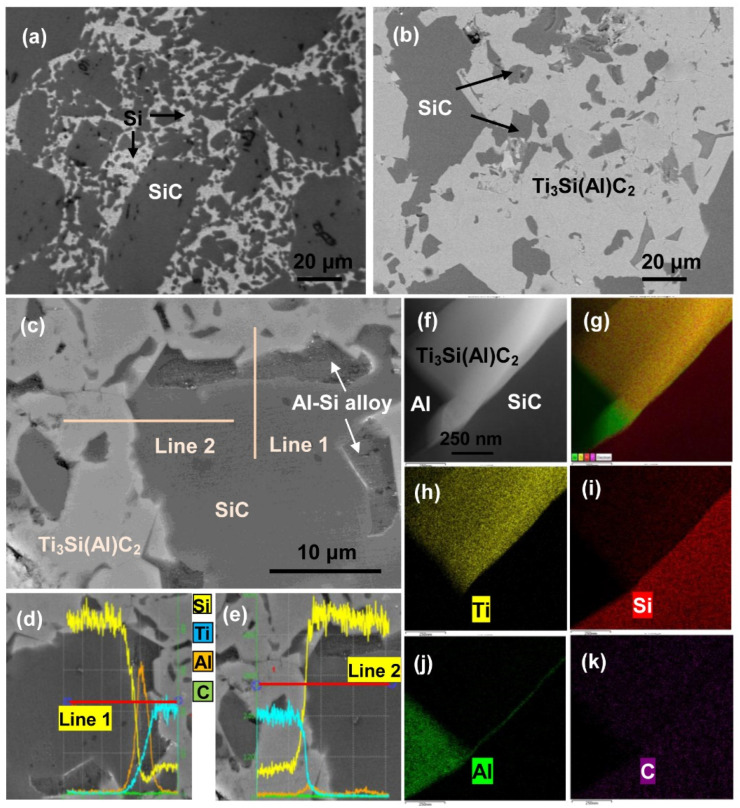
SEM images of SiC base ceramic, (**a**) the raw SiC ceramic, (**b**,**c**) SiC ceramic infiltrated with Al base alloy at 900 °C for 6 h, (**d**,**e**) EDS line scanning results of Line 1 and Line 2. (**f**–**k**) TEM and EDS mapping images of sample infiltrated at 900 °C for 6 h.

SEM, EDS, and TEM analyses were performed on SiC ceramics infiltrated by Al base alloy at 900 °C for 6 h to investigate the in situ synthesis process of Ti_3_Si(Al)C_2_. From Figure 10a,b, and Table 1, it can be seen that Ti_3_Si(Al)C_2_ is generated in situ on the SiC ceramic during infiltration. The size of the SiC grains gradually decreased as the Ti_3_Si(Al)C_2_ grains grew. A higher magnification image of the sample is shown in Figure 10), where a white Ti_3_Si(Al)C_2_ phase can be observed near a grey SiC phase and a black Al–Si alloy structure. The EDS line scanning analyses in Figure 10d,e indicate that a transition layer of Al base alloy exists on the SiC phase, from which we can infer that molten Al penetrates into the SiC ceramic samples. According to the distributions of Ti, Si, Al, and C (Figure 10h–k, respectively), it can be seen that white phase mainly contains Ti, Si, C, and a small amount of Al, thus corresponding to the Ti_3_Si(Al)C_2_ phase. The lower left corner is mainly Al, whereas the lower right corner contains Si and C; therefore, it is inferred that the lower left is Al and the lower right is SiC. Al distribute in the interface between Ti_3_Si(Al)C_2_ and SiC, which further illustrates that Ti_3_Si(Al)C_2_ is formed by the reaction of Ti with Al and SiC. At the same time, it can be deduced that Al atoms were substituted into a small number of Si sites in Ti_3_SiC_2_, thus forming Ti_3_Si(Al)C_2_. 

**Figure 11:**
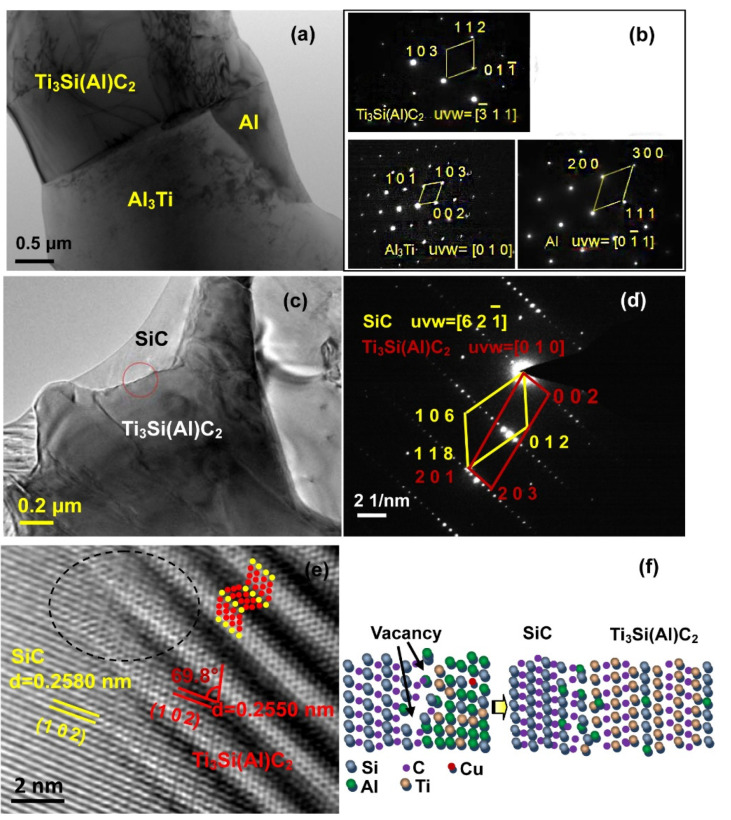
TEM investigations of the infiltrated sample: (**a**,**b**) SiC ceramic infiltrated with Al base alloy at 900 °C for 0.5 h, (**c**–**e**) SiC ceramic infiltrated with Al base alloy at 900 °C for 6 h, (**f**) schematic diagram of Ti_3_Si(Al)C_2_ formation process.

The above analysis further confirms that neither TiC (TiC_x_) nor Ti_5_Si_3_ (Ti_5_Si_3_C_x_) are present in the interface between Ti_3_Si(Al)C_2_ and SiC. Hence, to elucidate the synthesis process, SiC ceramics infiltrated with Al base alloy at 900 °C for 0.5 h were examined by TEM and SAED (Figure 11a). It can be seen that Ti_3_Si(Al)C_2_ coexists with Al_3_Ti and Al phases. Due to the short infiltration time, only a small amount of Ti_3_Si(Al)C_2_ had formed. Hence, unreacted Al and Ti crystallized to the Al and Al_3_Ti phase when cooling after the infiltration process, showing that the molten Al base alloy surrounds and reacts with SiC during melt infiltration. In order to ascertain the growth mechanism, TEM investigations of the interface between SiC and Ti_3_Si(Al)C_2_ were carried out. The low magnification TEM image in Figure 11c shows SiC grains surrounded by Ti_3_Si(Al)C_2_ particles, which were further confirmed by the corresponding SAED pattern in Figure 11d. The SiC phase belongs to the space group *P*6_3_/*mmc* (ICDD PDF 48-0708), and its lattice parameters are *a* = 0.3079 nm and *c* = 2.0147 nm. Ti_3_Si(Al)C_2_ also belongs to the space group *P*6_3_/*mmc* (ICDD PDF 48-1826), and its lattice parameters are *a* = 0.3068 nm and *c* = 1.767 nm. Hence, SiC and Ti_3_Si(Al)C_2_ both have a hexagonal crystal unit cell with similar lattice parameters. Figure 11e shows that the lattice at the interface is continuous and matches well, and only existing a few of vacancies. According to Figure 9b, it can be inferred that molten Al lowers the Si–C bonding strength and decompose SiC [28]. This was similarly found in the work of Fan [6]. He et al. [29] also found that when SiC is in contact with molten Ag–Cu–Ti alloy, it can dissolve into Si and C, and then react with active Ti species. The vacancies and special orientation relationship between SiC and Ti_3_SiC_2_ reduce the interfacial energy, which is beneficial for the formation of Ti_3_Si(Al)C_2_. We can further infer that Ti_3_Si(Al)C_2_ is generated on SiC by the infiltration of molten Al base alloy.

During the infiltration procedure, molten Al base alloy penetrates into the SiC ceramic, first by dissolving Si located in the gaps of SiC particles, and then forming Al-Si eutectic with low-melting-point, which constructed the channels for infiltration. Finally, the Al base alloy wets and reacts with the SiC ceramic. The identified Ti_3_Si(Al)C_2_ formation process is shown in Figure 11f. Al, Ti, Si, and a small amount of Cu atoms surround the SiC particles. Al atoms decompose the SiC, creating some vacancies, which can be seen from Figure 9b and Figure 11e. In addition, the higher affinity of Ti to C than to Al, and the close structural relationships between SiC and Ti_3_Si(Al)C_2,_ enable the in situ growth of Ti_3_Si(Al)C_2_ on SiC [30]. Therefore, the formation of Ti_3_Si(Al)C_2_ can be summarized by Equation (1):

(1) $$2\mathrm{SiC}+3\mathrm{Ti}+\mathrm{Al}\to Ti_{3}{\mathrm{Si}(\mathrm{Al})C}_{2}+\mathrm{Si}$$

It can be seen that Al not only opens the infiltration channel by dissolving the Si in the gaps of SiC, but also rapidly transports Ti to SiC due to the low melting point. Moreover, the molten Al alloy dissolves the Si released by Reaction (1). When Si is saturated in the Al melt, excess Si will precipitate. At the same time, Reaction (1) will be suppressed. This result corresponds with the results of infiltration for a long time. Indeed, in our previous work [28], we demonstrated that excessive Si can completely inhibit the formation of Ti_3_Si(Al)C_2_.

**Figure 12:**
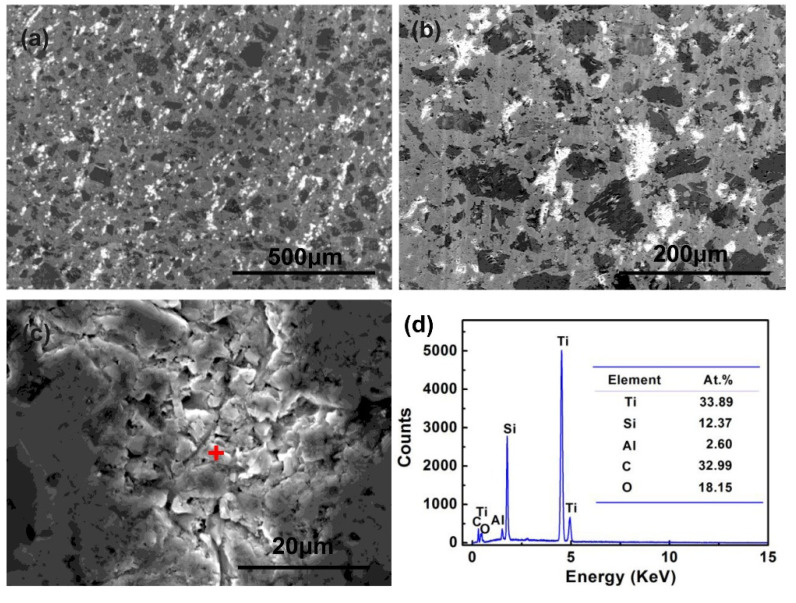
(**a**–**c**) SEM images of SiC ceramic infiltrated at 1000 °C for 4 h with different magnifications, and (**d**) EDS analysis of the point in (**c**).

Figure 12a–c shows morphologies of the SiC ceramic infiltrated with Al base alloy at 1000 °C for 4 h. The surface of the sample presented bright white particles, with black SiC phases and grey Ti_3_Si(Al)C_2_ phases. Figure 12c,d shows a high magnification image and EDS analysis of the white bright phases. The agglomerate Ti_3_Si(Al)C_2_ was dissociated into small blocks, and contained 33.89 at.% Ti and 32.99 at.% C. Combined with the XRD patterns in Figure 2, the small blocks were inferred to be TiC. From Figure 5 and Figure 12, it is suggested that Ti_3_Si(Al)C_2_ was unstable at 1000 °C; prolonged infiltration at 1000 °C resulted in the decomposition of Ti_3_Si(Al)C_2_.

**Figure 13:**
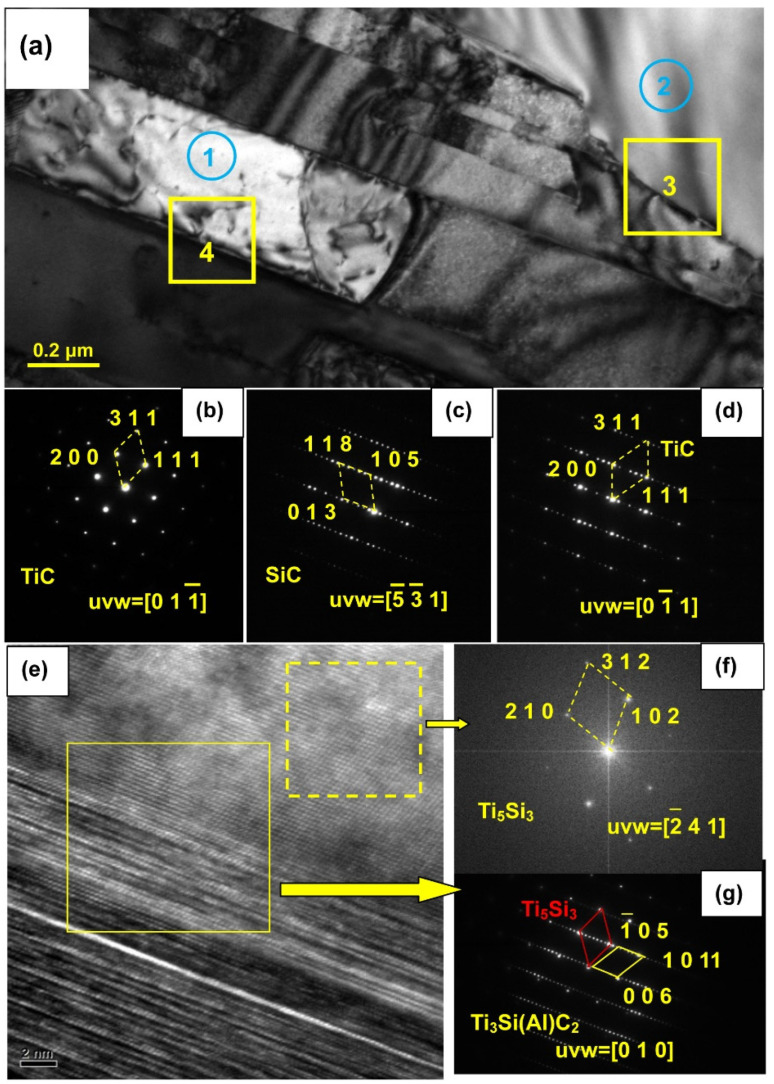
HRTEM images (**a**) and SAED patterns of the SiC sample infiltrated with Al alloy at 1000 °C for 6 h, (**b**–**d**) SAED patterns of area 1–3, (**e**–**g**) HRTEM and SAED of area 4.

Figure 13 shows HRTEM images and the SAED pattern of the sample infiltrated at 1000 °C for 6 h, which allows identification of the phases in areas 1–4. The general view shows that the banded structure is TiC. Figure 13b proves that the electron beam is parallel to the [01$\overset{-}{1}$] axis. Area 2 is SiC, as seen from the SAED micrograph in Figure 13c. Area 3 contains SiC (from area 2) and TiC. Moreover, some Ti_3_Si(Al)C_2_ and Ti_5_Si_3_ can be identified, from Figure 13e–g. Therefore, it can be inferred that Reactions (2) and (3) occur at 1000 °C. In the 1000 °C melt infiltration experiment, SiC ceramic surface oxidation film will be removed by deoxidization. Al base alloy infiltrated SiC materials because the Al base alloy wetted with SiC ceramic dissolved Si existing in the gaps of SiC particles. Ti atoms absorbed around the SiC particles, under the condition of high temperatures of 1000 °C are more active. Ti atoms directly reacted with SiC and generated a lot of TiC. A small amount of Ti_3_Si(Al)C_2_ is also obtained. Ti_3_SiC_2_ and Ti_3_Si(Al)C_2_ are not thermodynamically stable at high temperatures [31]. With the extension of the infiltration time, the TiC and a small amount of Ti_5_Si_3_ were produced by the reaction between the active Ti atoms and the small amount of Ti_3_Si(Al)C_2_. Due to the small amount of Ti_5_Si_3_, it was rarely detected in the XRD phase analysis.

(2) $$3Ti_{3}SiC_{2}+2Ti=6TiC+Ti_{5}Si_{3}$$

(3) SiC+Ti=TiC+Si

### 3.3. Flexural Behaviours

**Figure 14:**
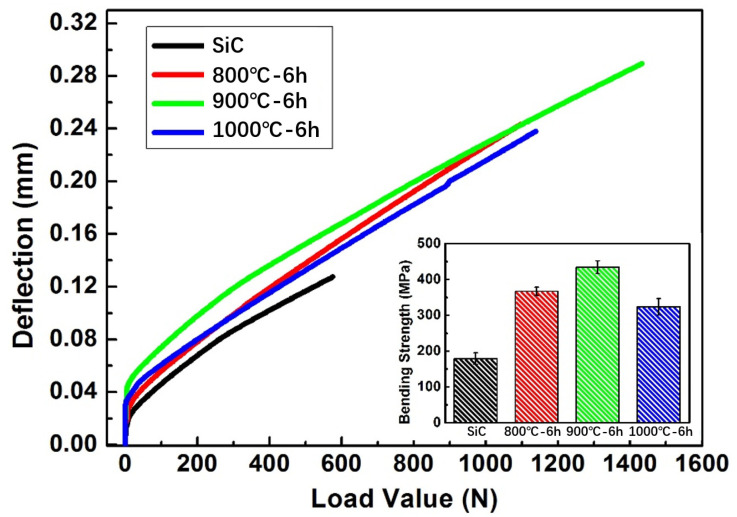
Load-deflection curves and three-point bending strength of SiC ceramics and SiC infiltrated at 800, 900, and 1000 °C for 6 h.

**Figure 15:**
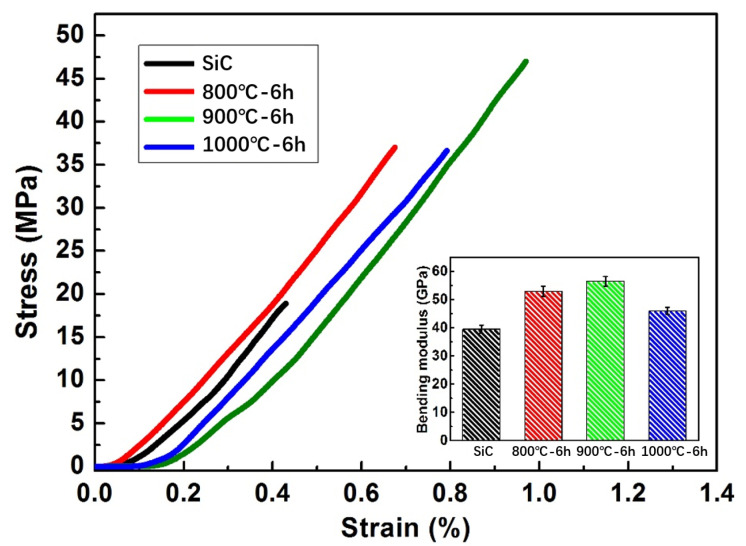
Bending strain-stress curves and bending modulus of SiC ceramics and SiC infiltrated at 800, 900, and 1000 °C for 6 h.

From the above analysis, the microstructure and phase composition of the reaction layer on the surface of SiC ceramic sample are different for the samples infiltrated at different temperatures. The inner of SiC ceramic sample consists of Al alloy and SiC, which was reported in our previous work [32]. Figure 14 shows the load-deflection curves and three-point-bending strength of the samples. The curves show that the sample infiltrated at 900 °C for 6 h exhibited the largest load and deflection values. Compared to the raw SiC ceramic and samples infiltrated at 800 and 1000 °C, the three-point strength of samples infiltrated at 900 °C was obviously improved. Figure 15 shows the bending stress-strain curves and bending modulus of the original SiC ceramic and SiC samples infiltrated with the Al base alloy at 800–1000 °C for 6 h. It can be seen that the sample infiltrated at 900 °C for 6 h exhibited the largest stress and strain values. Moreover, the bending modulus is also higher than SiC ceramic and samples infiltrated at 800 and 1000 °C.

The increase in the strength could be attributed to the infiltrated Al alloy and the newly formed Ti_3_Si(Al)C_2_ phases. The hardness of Al alloy is lower than that of Si and SiC, but the toughness of Al alloy is stronger than that of Si and SiC. The Al base alloy is stronger and tougher than Si and SiC. After infiltration, the Al alloy dissolved Si in the SiC particles gap and made close contact with SiC. Under the loading condition, the ductile fracture of Al alloy occurred instead of the brittle fracture of Si. In addition, due to the special layered structure and low hardness of Ti_3_Si(Al)C_2_, under the action of external force, Ti_3_Si(Al)C_2_ would be twisted, drawn, and layered, which consumed the energy of crack propagation, effectively suppressed crack propagation and thus improved the strength.

The formation of the Ti_3_Si(Al)C_2_ reaction layer on the SiC ceramic, and the samples infiltrated at 900 °C showed remarkable advantages over those infiltrated at 800 and 1000 °C. Ti_3_Si(Al)C_2_ is a soft ceramic with a hardness of about 500 HV, which is much lower than that of SiC and TiC. Furthermore, Ti_3_Si(Al)C_2_ had in situ synthesis on SiC and formed a compact composite reaction layer. Similar lattice parameters and well matched interface between Ti_3_Si(Al)C_2_ and SiC contribute to the enhancement of mechanical properties. Therefore, the strength of the samples infiltrated by at 900 °C is higher than that at 800 and 1000 °C.

## 4. Conclusions

In this paper, the effects of infiltration temperature on the microstructure, phase composition, and mechanical properties of SiC base ceramic infiltrated by Al base alloy at atmosphere circumstances were investigated. Infiltration at a relatively low temperature (e.g., 800 °C) did not trigger the formation of Ti_3_Si(Al)C_2_; however, the Ti_3_Si(Al)C_2_ phase became unstable and decomposed into TiC and Ti_5_Si_3_ when the composites were infiltrated at 1000 °C. A reaction layer mainly containing Ti_3_Si(Al)C_2_ formed out of SiC by Al base alloy reacting with the Si component in SiC ceramic when infiltrated at 900 °C, which was much lower than the traditional sintering temperature (usually above 1400°C) of Ti_3_SiC_2_. SiC and Ti_3_Si(Al)C_2_ shared a coherent interfacial structure and well- matched lattice. Thus, the infiltration of Al alloy and formation of Ti_3_Si(Al)C_2_ enhanced the flexural modulus and three-point bending strength.

## Figures and Tables

**Table 1:** EDS analysis of the different microstructures in Figure 10c.

Main Phase	Chemical Elements (at.%)				
	Al	Si	Ti	C	Cu
Ti_3_Si(Al)C_2_	1.24	14.96	47.37	36.43	0.00
SiC	0.42	48.03	0.00	51.55	0.00
Al-Si alloy	52.78	14.58	0.00	30.71	0.92
